# Supplementary material for: Novel Driver Strength Index highlights important cancer genes in TCGA PanCanAtlas patients
Source: PeerJ. 2022 Aug 11;10:e13860. doi: 10.7717/peerj.13860 (PMC9375969; doi:10.7717/peerj.13860)
Supplement: Supplemental Information 2 [file peerj-10-13860-s002.zip › COHORTS/genes plots/detailed/2021_8_16_14_9_distribution_events_detailed_males_47.pdf]

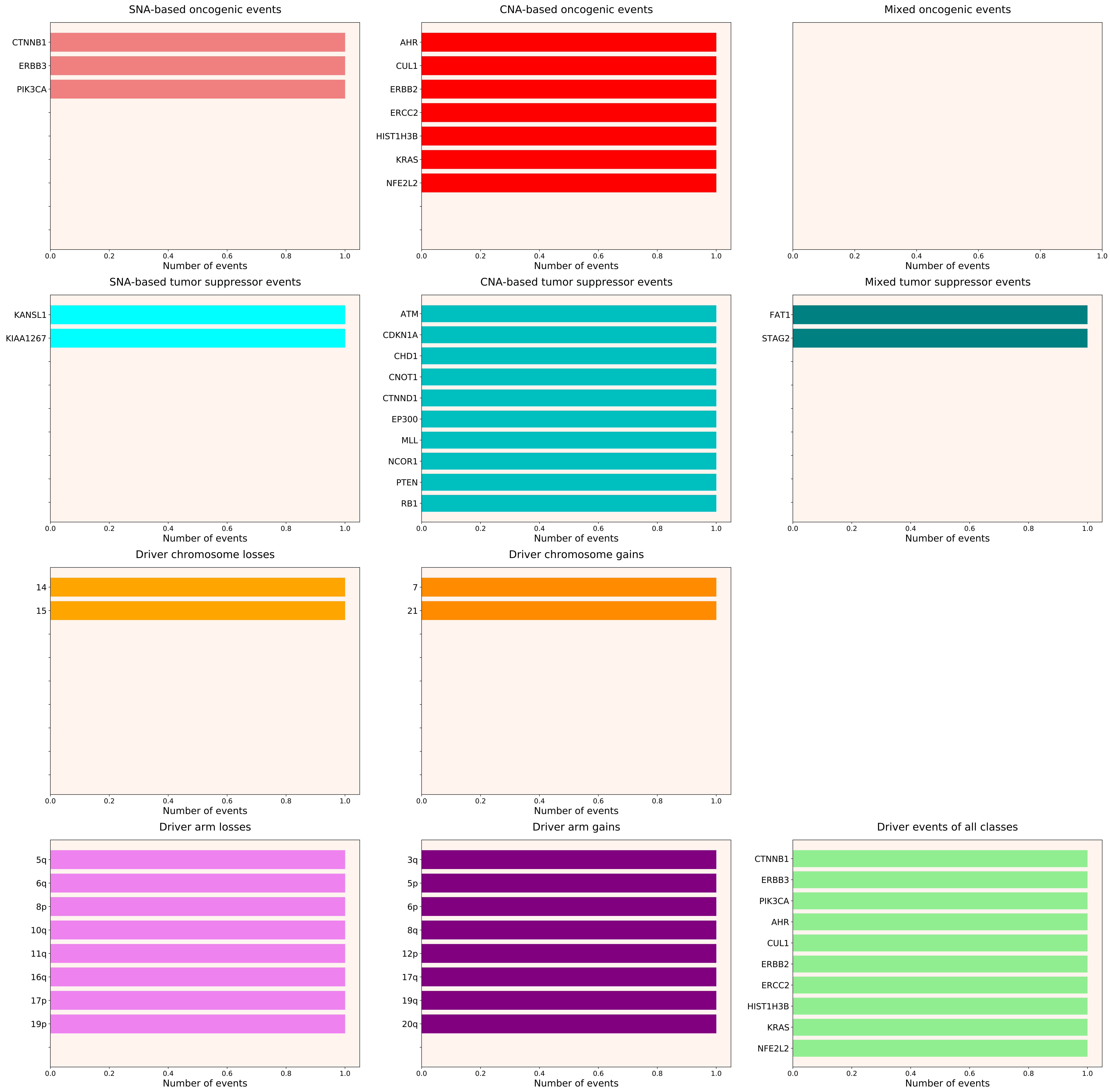

SNA-based oncogenic events
CTNNB1
ERBB3
PIK3CA
0.0
0.2
0.4
0.6
0.8
1.0
Number of events
CNA-based oncogenic events
AHR
CUL1
ERBB2
ERCC2
HIST1H3B
KRAS
NFE2L2
Number of events
Mixed oncogenic events
Number of events
SNA-based tumor suppressor events
KANSL1
KIAA1267
Number of events
CNA-based tumor suppressor events
ATM
CDKN1A
CHD1
CNOT1
CTNND1
EP300
MLL
NCOR1
PTEN
RB1
Number of events
Mixed tumor suppressor events
FAT1
STAG2
Number of events
Driver chromosome losses
14
15
Number of events
Driver chromosome gains
7
21
Number of events
Driver arm losses
5q
6q
8p
10q
11q
16q
17p
19p
Number of events
Driver arm gains
3q
5p
6p
8q
12p
17q
19q
20q
Number of events
Driver events of all classes
CTNNB1
ERBB3
PIK3CA
AHR
CUL1
ERBB2
ERCC2
HIST1H3B
KRAS
NFE2L2
Number of events
